# Esthetic Surgery of the Chin in Cis- and Transgender Patients—Application of T-Genioplasty vs. Single-Piece Segment Lateralization

**DOI:** 10.3390/medicina60010139

**Published:** 2024-01-11

**Authors:** Rafał Pokrowiecki, Barbora Šufliarsky, Maciej Jagielak

**Affiliations:** 1 Prive Esthetic and Facial Feminization Surgery Centre, 02-640 Warsaw, Poland; 2 Department of Oral and Maxillofacial Surgery, Faculty of Medicine, Comenius University in Bratislava and University Hospital, 81372 Bratislava, Slovakia; barbora.sufliarsky@gmail.com; 3 Orthognatic, Private Practice, 05-090 Warsaw, Poland; recepcja@ortognatyka.pl

**Keywords:** facial feminization, gender-affirmation surgery, genioplasty, plastic surgery, craniofacial surgery, orthognathic surgery

## Abstract

*Background and Objectives:* Correction of lower face asymmetry still remains challenging in maxillofacial surgery. This report describes techniques for the lateral transposition of the symphyseal segment to restore lower face symmetry while maintaining gender-related features in cis- and transgender patients. *Materials and Methods:* A retrospective review of medical records of 31 patients who attended for esthetic corrective surgery after orthodontic camouflage or orthognathic treatment, or during facial feminization of the lower face between June 2021 and June 2023 was performed. *Result:* All patients underwent lateralization genioplasty (with or without advancement or setback), either with or without narrowing T-osteotomy supplemented with necessary procedures in order to obtain proper facial balance and desired esthetical effects, such as bichectomy, liposuction, and face and neck lift. The mean asymmetry of the chin was 5.15 mm and was surgically corrected either by single segment lateralization or T-shape narrowing genioplasty depending on the gender and esthetical requirements. No complications were reported. *Conclusions:* Lateral shift genioplasty serves as a powerful tool in primary and secondary corrective surgery for lower face asymmetry that maintains gender-specific facial features. It may serve either as an additive to orthodontic camouflage or a way to correct previous orthognathic surgery pitfalls. The surgeon performing esthetic genioplasty associated with gender-specific expectations must be trained in facelift and facial liposculpting techniques in order to provide the best results and properly choose the right procedures for the right patients.

## 1. Introduction

Esthetic considerations of the lower third of the face are one of the most commonly encountered problems among cis- and transgender patients. The chin, as a central part of the face, and the nose are responsible for one to be considered symmetrical or not. In esthetical perception, the nose is regarded as a “chief seat” of the face. Nevertheless, chin projection, shape, and interplay with proportions of the whole face play equal roles in facial esthetic surgery.

In clinical reality, there are numerous causes of lower face asymmetry resulting from congenital factors, malocclusion, trauma, incorrect dental treatment, functional factors, or tumors. However, patients being referred for revision surgery or supplemental procedures that focus on the chin symmetrization have usually had either camouflage orthodontics without jaw surgery and/or esthetic dental camouflage or have undergone orthodontic or plastic surgery procedures that failed to fully resolve the initial problem. The second issue associated with lower face esthetics is rooted in gender-related requirements. Cis- and transgender women usually wish to have a heart-shaped face with a smaller nose and tapered chin [1] Biological and transgender males prefer to have sharp, long, straight noses, as well as prominent jawlines and gonial angles [2]. Consequently, the position and shape of the chin determine the thickness and projection of the overlying soft tissues, which play crucial roles in facial esthetics. The relationship between soft and hard tissues may vary between males and females [3]. It has a significant impact on the individualized treatment plans as desires and expectations may differ between cis- and transgender individuals.

Sliding genioplasty is an established surgical technique for the correction of sagittal dental skeletal malocclusion. However, its application as corrective surgery of lower face asymmetry in complicated cases is rarely reported. This study encompasses the utility of genioplasty techniques in chin symmetrization with regard to gender-identity requirements along with supplemental procedures involving surrounding soft tissues for the best esthetic results.

## 2. Materials and Methods

A retrospective review of medical records of patients who attended for esthetic corrective surgery of the lower face between June 2021 and June 2023 was conducted. Inclusion criteria were patients who complained of chin asymmetry followed by orthodontic/orthognathic procedures and/or transgender women who attended for feminization surgery of the lower face. Exclusion criteria included incomplete files and adolescents. This study aimed to analyze the previously performed procedures in the lower face performed elsewhere, complications, and reasons for revision surgery. This study was conducted in accordance with the principles of Helsinki. Written informed consent was acquired from the individuals whose pictures are presented within this manuscript.

## 3. Results

**Figure 1:**
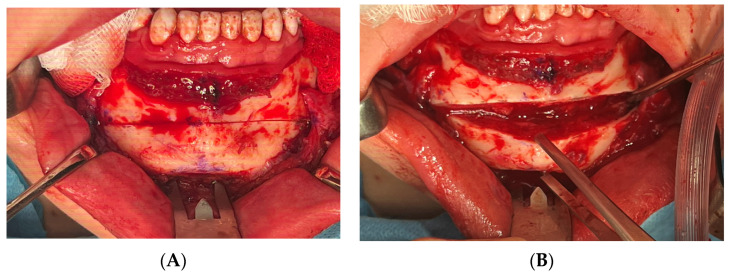

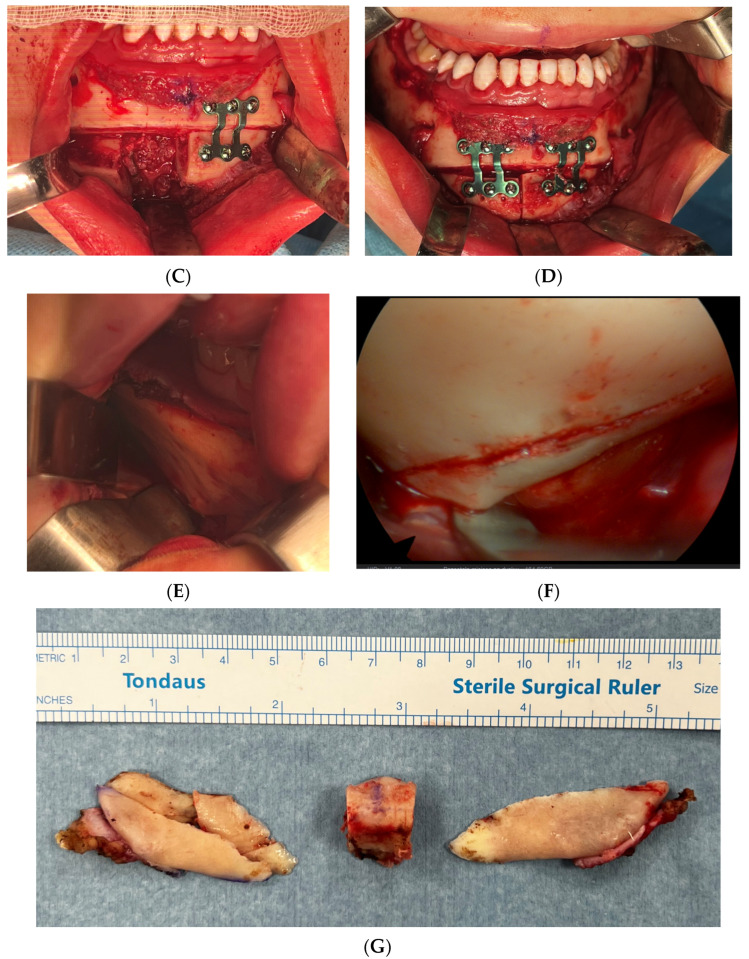
Intraoperative photographs of horizontal T-shape genioplasty, which was most commonly performed in feminization surgery procedures. (**A**,**B**) Horizontal osteotomy and bone segment detachment. In cases with lateralization, bone segment was transpositioned contra-laterally to the deviation, and bone steps and irregularities were burred down with diamond burr. (**C**,**D**) Bone segment sectioned in the midline into two pieces with resection of the central bone block (Video S1). Bone segments were stabilized with 2.0 mm titanium plates (ChM, Poland) (in this particular case, 3 mm setback was necessary to reduce protruding chin but no deviation was diagnosed). (**E**,**F**) V-shape surgery for the purposes of mandible feminization was supplemented with endoscopy-assisted bilateral resection of the mandibular angles, which was performed in a way resembling bilateral sagittal split osteotomy with additional sections and osteoplasty that enabled sufficient narrowing of the mandible. (**G**) Photograph of the resected mandibular angles along with partial resection of the masseter and central genial bone block.

**Figure 2:**
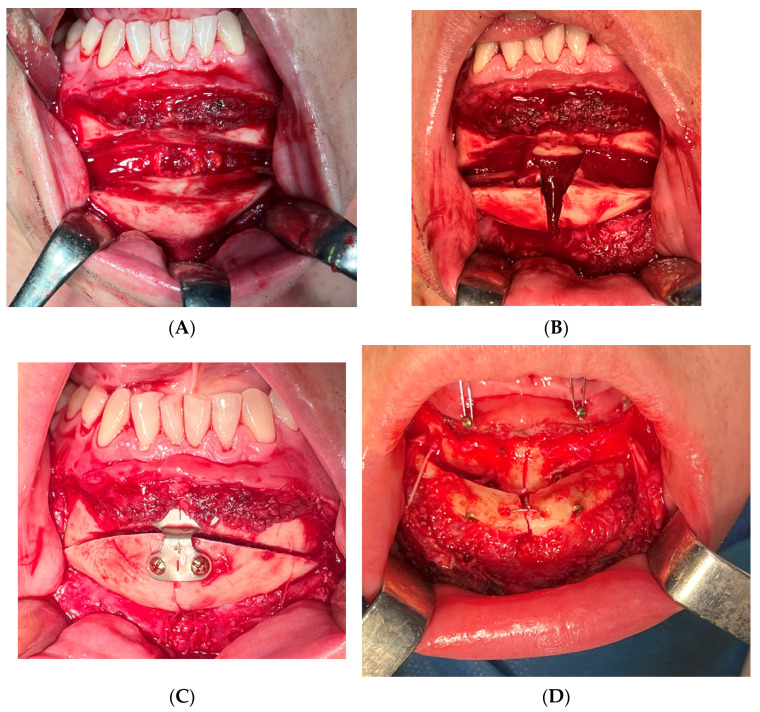
Examples of T-shape genioplasty variants other than horizontal performed in the presented material. (**A**–**C**) M-genioplasty. In this case, frontal advancement of 4 mm was necessary and bone segments were stabilized with single 2.0 titanium plate. (**D**) T-shape genioplasty originally described by Greme and Blenkisopp (1990) modified by Jagielak’s application of 2 compression screws (2.0 × 20 mm, Medartis, CHF), which allowed for advancements larger than 10 mm with preservation of bone continuity and stable fixation without necessity of mandibular sagittal split osteotomy.

**Figure 3:**
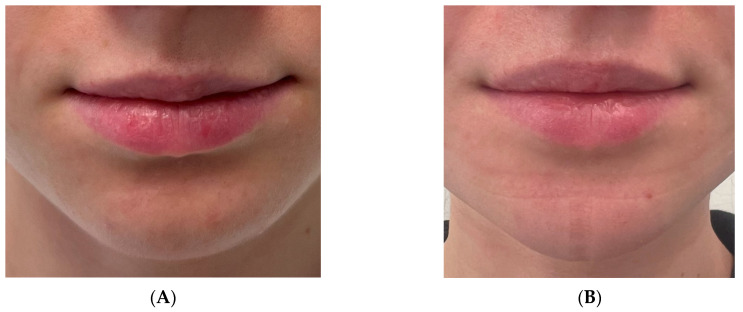

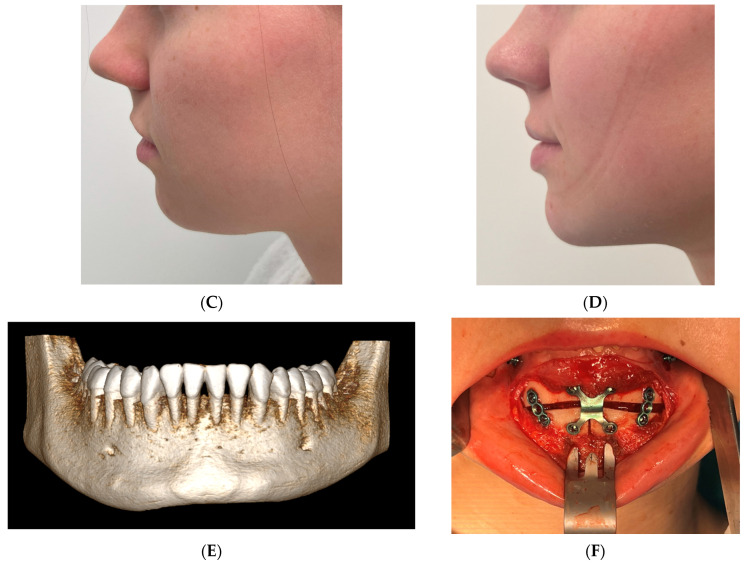
Example of 23-year-old cis-gender female who complained of vertical asymmetry and receded chin. In this case, T-shape genioplasty with advancement supplemented with submental liposuction and non-surgical neck lift (VASER) was performed, which restored symmetry and feminine and youthful look of the lower third. (**A**) Frontal photograph before surgery, (**B**) frontal photograph after the surgery. (**C**) Lateral view before and (**D**) after the surgery. (**E**) Three-dimensional reconstruction showing vertical asymmetry. (**F**) Interoperative view of the reduced, transpositioned, and advanced bone segments (ChM, Lewickie, Poland).

**Figure 4:**
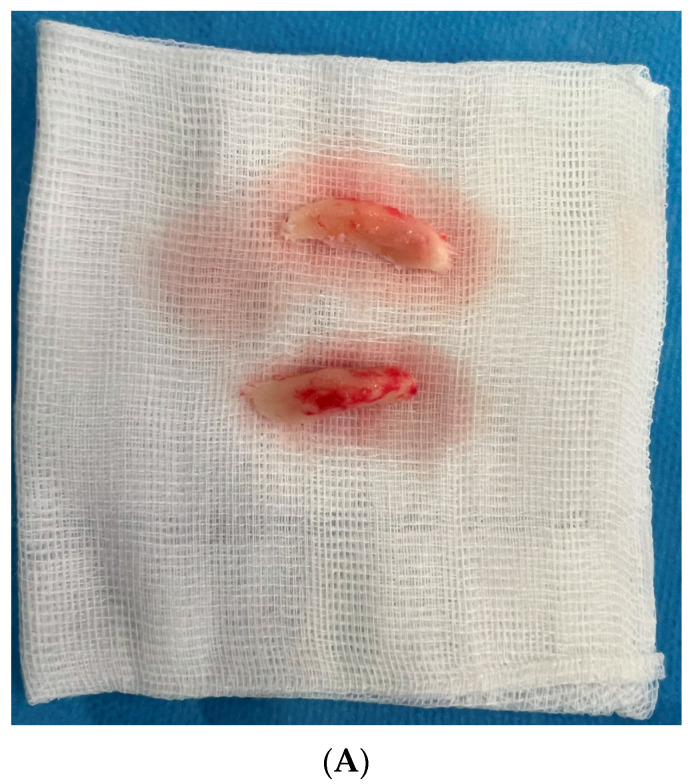

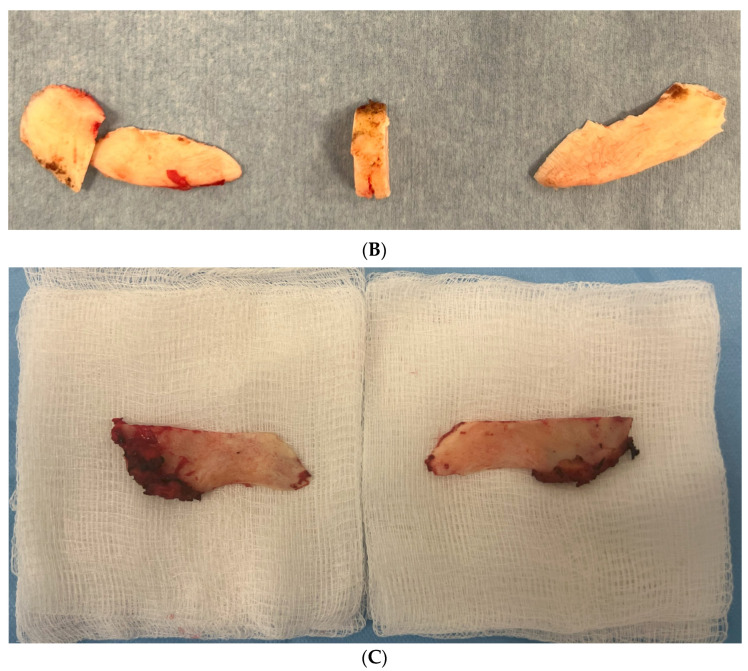
Intraoperative photographs of the resected mandibular angles in different patients. (**A**) A very conservative resection of the lateral aspects of the gonial angles performed in cisgender woman. (**B**) Intermediate resection involving gonial angles and partial ramus and mandibular body (note 5 mm central block of the bone from the T-genioplasty) (cisgender woman with square lower third), (**C**) extended resection of the angles involving cortical bone of the ramus and body and partial resection of the pterygoid–masseteric sling (transgender woman—facial feminization procedure).

A total of 31 patients were referred for midline chin osteotomy between December 2021 and November 2023. Common complaints reported by patients were chin asymmetry and/or hypoplasia after orthodontic camouflage (n = 20) or orthognathic surgery (n = 6). Eleven patients were referred for facial feminization of the lower third as a part of their male-to-female transition procedures. Nineteen patients presented significant chin deviation which required symmetrization surgery. The average chin asymmetry, concerning the midline, ranged from 3.46 mm to 8.45 mm (average 5.15 mm) (Table 1). Fifteen patients decided on feminizing chin mandibuloplasty (twelve transgender females and three cisgender females). In these patients, variants of T-shape genioplasty were performed (Table 2 and Table 3) (Figure 1, Figure 2, Figure 3 and Figure 4) (Video S1). In cases where the deviation ranged up to 4.5 mm, no lateralization of the segment was required (n = 11) (Figure 3). Additional lateralization of the chin after T-genioplasty was required in cis- and transgender females when the deviation was >4.5 mm (n = 7) (Figure 3).

Also, additional endoscopy-assisted feminization surgery of the mandible (V-shape ostectomy) was performed in 12 cases (three cisgender females, nine transgender females) (Table 2) (Figure 1). In cisgender females, conservative ostectomy was performed, while transgender patients required more aggressive ostectomy with partial resection of the masseter (Figure 4).

**Figure 5:**
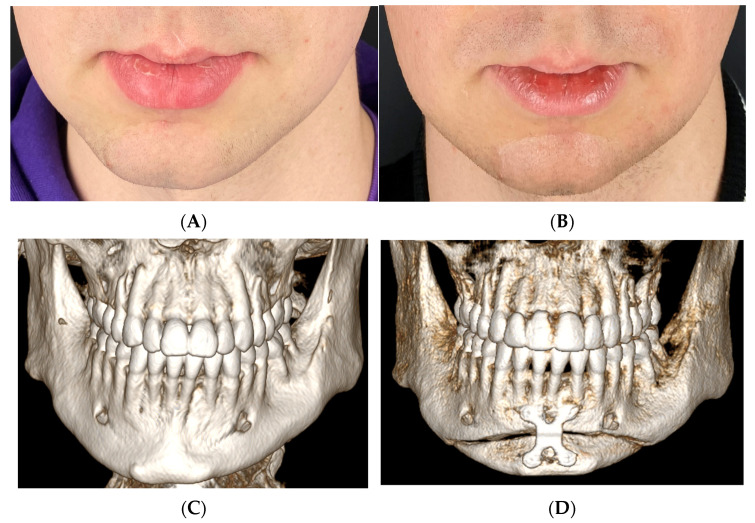
A 27-year-old male who referred for chin symmetrization after orthodontic treatment and bimaxillary surgery performed elsewhere. He did not opt for corrective orthodontic surgery and revision orthognathic surgery. In this case, segmental chin lateralization with 3 mm setback and lower border recontouring were performed.

Five males desired symmetrization and single-segment lateral shift genioplasty was performed followed by osteoplasty/ostectomy of the residual bone at the lower border of the mandible. In three males, additional masculinization of the mandibular angles with 3D Virtual Surgical Planning and custom implants was performed (Figure 4 and Figure 5).

In order to obtain optimal esthetic results, preserve skin elasticity, and reduce fat tissue from the chin and neck, additional procedures were performed. These included bichectomy (n = 9), liposuction (n = 12), Vibration Amplification of Sound Energy at Resonance liposuction (Vaser, Sound Surgical Technologies, Louisville, CO, USA) (n = 10), central neck lift (n = 5), which were performed simultaneously. Six patients required a supplementary facelift, which was performed simultaneously or 3–6 months after bone recontouring procedures. The techniques used were patient-specific and included Minimal Access Cranial Suspension (MACS) lift (n = 1), deep-plane face and neck lift (n = 4), and endoscopic assisted full facelift (ponytail facelift) (n = 2) (Table 4). No complications after the surgery were reported apart from transient lower lip numbness, which resolved after 3–6 months in all presented cases.

## 4. Discussion

This study described a simplified midline osteotomy for the correction of facial esthetics in a group of patients complaining about lower third asymmetry following orthodontic compensation. Midline osteotomy techniques of the chin as adjuvant surgery have been reported since 1960. The first osteotomies were based on resection of the midline single bone block and mandible constriction in the treatment of dentofacial deformities. They have been reported to be effective and require significant postoperative downtime. A 10 mm bone segment resection is considered safe for mandibular constriction, without periodontal or temporomandibular joint function contraindications [4,5]. Modified T-shape osteotomy for the correction of asymmetry and advancement was further described by Grime and Bleinkinsopp in 1990 [6]. Midline osteotomy is still considered to be an adjuvant in orthognathic surgery where transverse discrepancy must be corrected [7].

In our opinion, the maximal width of 10 mm of resected chin midline segment is accurate from the point of view of classic orthognathic surgery and not exactly in transgender facial surgery. In our study, resection of the chin segment was followed by its lateral reposition (asymmetries less than 4.5 mm) or followed by T-shape genioplasty based on central bone block resection. The latter was performed in cis- and transgender women and hence, the volume of the bone block corresponded to the deviation extent and feminizing desires of the patients. In our material, blocks wider than 10 mm may be safely resected in feminizing procedures without any harm to periodontal tissues and jawline projection (Figure 1). However, lateral bone fragments should not be totally detached from the surrounding tissues due to the risk of resorption.

There are numerous techniques for chin augmentation osteotomies. In our study, we performed horizontal T-shape osteotomies (n = 13). Two of them were modified T-shape osteotomies with advancement described by Grime and Blenkisopp (1990) [6] stabilized with centrally placed bone wire and two 20 × 2.0 mm compression screws (Jagielak’s modification) (Table 3) (Figure 2). This technique allows for the correction of asymmetry and two-stage advancement, which, in some cases, is troublesome to obtain with stock titanium plates. Most of the cases did not require significant advancements and hence, rigid fixation plates and screws were applied after performing chin narrowing and shift correcting the asymmetry. There were two cases where M-osteotomy was performed according to the technique of Lee et al. (2018) where no vertical augmentation was indicated [8]. Another two cases required modified M-osteotomy of the chin with additional advancement according to [9].

In the presented study, cisgender females had usually been previously treated orthodontically followed by cosmetic dentistry procedures such as porcelain veneers or composite work-up (bonding). Among these patients, symmetrization of the chin was a common problem. Patients were not informed by their orthodontist about facial asymmetry and indications for orthognathic surgery in the first place. Patients refused to undergo secondary orthodontic treatment and subsequent orthognathic surgery. Therefore, they were qualified for esthetic corrective treatment either with single-segment lateral shift mandibuloplasty or T-shape genioplasty.

On the contrary, transgender females have usually chosen cosmetic dentistry camouflage instead of orthodontic treatment before scheduling an appointment for corrective surgery. In transgender females, it is commonly seen that they choose the “fast” esthetic effect as transition is a long process and they usually choose shorter ways to enhance feminine features from the “esthetic” point of view but not necessarily in the functional aspect (malocclusion, dentofacial abnormalities requiring orthognathic surgery, etc.). Orthodontic treatment usually lasts 3–4 years and such a period of time is frequently unacceptable for transgender women. Moreover, as facial feminization surgery is not usually covered by national health insurance in many countries, patients seeking gender confirmation procedures try to save as many funds as possible to be able to pursue other FFS procedures. Last but not least, transgender oral healthcare still needs development and adaptation for the special needs of the transgender community. Hormone replacement therapy (HRT) (Estradiol) may affect or even impede orthodontic treatment or dental implant therapy, hence requiring reduction or modification of the hormone dosage. Therefore, patients usually choose a treatment plan that does not require a decrease in HRT [10]. 

Transgender women usually underwent reduction T-genioplasty and endoscope-assisted mandibular angle reduction. There were two cisgender women who wanted an angle reduction procedure along with chin correction. In these cases, more conservative resection was performed when compared to transgender patients so as not to over-resect the angles (Figure 4A).

**Figure 6:**
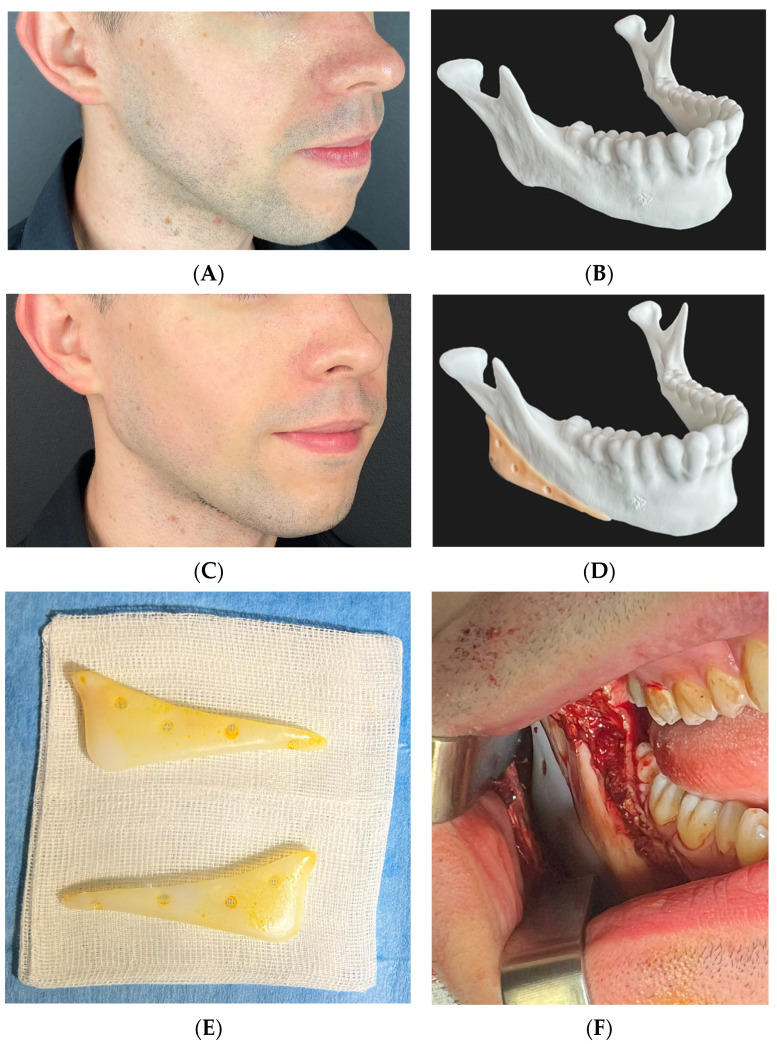
A 26-year-old male after orthodontic camouflage. He did not opt for corrective orthodontic surgery and revision orthognathic surgery. Patient wanted to correct receded chin and to masculinize hypoplastic mandibular angles which were caused by gonial deficiency resulting from class II malocclusion. Advancement genioplasty (5 mm forward, no asymmetry correction needed) and 3DVSP individual mandibular implants were performed. (**A**) Photograph before surgery, (**B**) 3D printed model of the mandible before surgery, (**C**) photograph after surgery, (**D**) mandible model with planned templates. (**E**) Implants (PTFE) before implantation, (**F**) intraoperative view of the right angle implant placed on the gonial angle and then stabilized with 5 × 2.0 mm titanium screws in order to provide stable fixation, preventing movement and hence encapsulation or infections.

Cisgender males were referred for simple correction of asymmetry and/or advancement after orthodontic treatment. They did not perform additional cosmetic dentistry such as veneers or bonding. Three patients wished to masculinize their faces by mandible augmentation with individual 3D custom implants (Figure 6). A total of six patients decided on symmetrization of the residual chin asymmetry that persisted after orthognathic surgery performed elsewhere. Chin symmetrization in cisgender males with or without masculinization (angle implants) was based on one-piece segment lateralization. It is advised not to perform T-genioplasty among cisgender males due to the feminization effect of such a procedure. Another interesting technique described by Raffaini and Sesenna in 1995 was based on the hemi-genioplasty technique for the correction of chin asymmetry [11]. However, as this technique is based on chin sagittal split osteotomy and lateralization on only the mobilized half of the chin, such a technique, in our opinion, is beneficial for cis- and transgender males as by that movement, the chin may be significantly wider. Moreover, such osteotomy does not provide possibilities for eventual advancement or setback, making this technique straightforward and simple, yet restricted to very specific indications [11]. In our study, we opted for full chin osteotomy and repositioning as it provided better control, soft tissue management, and advancement/setback when necessary.

This study also showed that asymmetries of the chin where the deviation is less than 4.5 mm may be corrected by single-segment chin lateralization and conservative mandibular shaving. Asymmetries more than 4.5 mm in cis- or transgender women may be sufficiently corrected with T-genioplasty. Resection of the central segment enables for a smooth shift of the chin and by narrowing, it offers a feminizing effect in both of these genders. It also reduces the risk of irregularities at the lower border of the mandibular body and provides a smooth transition between the shifted chin and the mandibular body.

Mandibular angle reduction was initially introduced into Korean facial surgery where patients desired less prominent mandibular angles. This surgery may be performed either as a Korean plastic surgery procedure alone or in combination with orthognathic surgery [12,13,14]. 

With time, this procedure was adapted for patients seeking feminization procedures, usually during the male-to-female transition [15,16,17]. In the study by Lee et al. (2023), a retroauricular approach for mandibular angle reduction was described [18]. In our opinion, this approach may be beneficial in cases where a simultaneous facelift is planned and two procedures may be performed through one access. However, in cases where genioplasty is planned and no facelift is performed, the classic intraoral approach through the oral vestibulum is still the safest; there is no risk of injury to the facial nerves and it leaves no scar on the skin. 

In our study, angle resection was performed simultaneously with T-shape genioplasty with endoscopic-assisted angle reduction as it provides direct vision and resection control. Endoscopic-assisted mandible osteoplasty for esthetic purposes was also described in the study by [19]. 

Similarly to Lee and Singh (2022), we observed the best feminizing effect after complex V-shape surgery consisting of angle reduction and chin feminization [16]. In cases requiring more aggressive resections, we also advocate for extended sagittal split ostectomy of the cortical bone of the ramus, angle, and mandibular body with partial excision of the masseter (Figure 4C) [15,20]. The distal border of the resection is preferably placed below the mental foramen with a smooth transition into the osteotomy of the chin. This reduces the risk of unwanted irregularities and enables conservative osteoplasty of the lower border and muscle attachment preservation. We did not perform total inferior border ostectomy as was described in the study by [21], where this technique was presented as superior to T-genioplasty [21]. The authors described total U-shape ostectomy of the whole mandibular complex en block in wider, more asymmetrical chins. None of our patients required this approach. Despite being useful, in certain cases, such a modification required the complete dissection of the muscle attachments from the inferior border. Detachment of the muscles generates a risk of postoperative chin ptosis, and hence we do not find justification for total muscle resections in esthetic surgery [22]. 

Facial masculinization surgery procedures are frequently performed in cis- and transgender males. Patients in transition from female to male constitute less than 10% of the transgender individuals. Both cis- and transgender males are groups of patients who usually desire to enhance their masculine features through chin and mandibular angle augmentation. In transgender males, masculinization of the lower third is the most commonly performed procedure and, along with recently described rib-graft Adam’s apple augmentation and testosterone HRT, is sufficient in reshaping the face [20,23].

While the chin may be masculinized either by advancement genioplasty or chin implants, mandibular angles require a different approach. As no osteotomy provides vertical augmentation in that particular anatomical area, the placement of stock or custom-made implants is a straightforward, safe, and effective procedure [24,25,26]. In our study, there were no transgender males; however, two cisgender males opted for masculinization, which shows the increasing interest of biological males in esthetic facial surgery procedures.

Soft tissue projection is crucial for the ultimate esthetic outcome. Surgical correction of the lower third requires addressing potential problems that may mask the osteoplastic procedures. These include buccal fat pad hypertrophy, appearing as visible bulging in the cheek area lateral to the nasolabial grooves, jowling, which may increase after angle resection and lower border marginectomy in certain cases, loose skin of the neck, platysma dehiscence, or contrarily, double chin and deep fat accumulation. Therefore, these conditions must be addressed before any bone recontouring, osteoplasty, or mandibular angle resection [16,27].

In younger individuals, facial liposculpting based on bichectomy and power-assisted liposuction may greatly enhance the V-shape goal of feminizing surgery. When no significant laxity of the skin is present, no additional procedures are indicated. In cases with minimal jowling, a MACS lift (MACSL) may be sufficient as it does not interfere in the area of the skin and is of great value in younger patients who do not want more aggressive facelift procedures at that age [28]. Full endoscopic facelift, currently known as “ponytail face lift”, is also of great utility. It is usually indicated in younger individuals without significant loose skin and greatly enhances feminizing procedures. Extended versions of the ponytail facelift may also be used in older patients [29]. However, significant jowling and excess skin present before reduction surgeries such as mandible angle resection or lower border ostectomy may require the traditional lower face and neck lift approach. A preferable technique is a deep-plane facelift as it focuses on re-draping the deeper layers of the face (SMAS and muscles) and not only re-draping and cutting off extra skin. Central neck lift, although a powerful tool in cases with platysma dehiscence and banding, causes accumulation of fat tissue. Power-assisted liposuction with central neck lift along with advancement genioplasty may dramatically re-drape, smoothen, and rejuvenate the neck area without a facelift approach. This approach is advocated in younger patients without significant jowls and skin laxity. These are better addressed by a classic deep-plane facelift or ponytail facelift where platysma tightening is performed at its distal aspect either by plication or transaction and platysma hammock techniques [29,30].

## 5. Conclusions

The presented study focused on the application of different variants of genioplasty for esthetic purposes in complicated orthodontic/orthognathic patients as well as transgender individuals. It described indications for lateralization of the chin in one segment or application of T-genioplasty variants in both correction of asymmetry and gender-confirmation facial surgery without revision orthodontic treatment nor orthognathic surgery based on bimaxillary surgery. For males, one-piece lateral shift genioplasty is advised. Residual bone at the lower mandible may be smoothened using a bone burr, preserving the male shape of the chin. For females with an asymmetry of <4.5 mm, a similar approach is advised, whereas asymmetries >4.5 mm require T-genioplasty to restore the feminine, triangular shape of the chin.

This is the first study describing procedures such as bichectomy, facial liposculpting, and face-lifting techniques as additive procedures necessary for a satisfying outcome. Endoscopic-assisted minimally invasive bone recontouring surgery procedures supplemented with 3D Virtual Surgical Planning and custom-made implants as well as properly addressing the surrounding soft tissues is undoubtedly the future of modern cosmetic surgery in both cis- and transgender patients.

## Figures and Tables

**Table 1:** Characteristics of the patients referred for the correction of the chin due to symmetry and/or gender-confirming features. F—cis female, F′—trans-female, M—male.

Patient	Gender	Age	Orthodontic	Orthognathic	Cosmetic Dentistry	Chin Deviation (mm)	Site
1	F	24	yes	no	no	5.87	R
2	M	32	yes	yes	no	6.72	R
3	F	39	yes	no	no	8.45	R
4	M	23	yes	yes	no	5.23	L
5	F′	31	yes	no	yes	3.46	R
6	M	32	yes	yes	no	4.67	R
7	M	53	yes	no	yes	7.12	L
8	F	23	yes	no	yes	4.31	L
9	M	53	yes	no	no	5.23	R
10	F	34	yes	no	yes	4.15	L
11	M	23	yes	no	no	4.56	R
12	F′	36	yes	yes	yes	6.64	L
13	F	21	yes	no	no	7.32	R
14	M	32	yes	no	no	6.57	R
15	F	38	yes	yes	yes	5.21	L
16	M	27	yes	no	no	3.96	R
17	F	22	yes	no	yes	5.12	R
18	M	19	yes	no	no	7.57	L
19	F	34	yes	yes	yes	7.43	R
20	F′	19	no	no	yes	1.1	L
21	F′	21	no	no	yes	0	N/A
22	F′	47	no	no	yes	0	N/A
23	F′	35	no	no	yes	2.1	R
24	F′	28	no	no	yes	0.5	L
25	F′	20	no	no	yes	0	N/A
26	F′	22	no	no	yes	0	N/A
27	F	27	no	no	no	0	N/A
28	F	35	yes	no	no	0	N/A
29	F′	42	no	no	yes	0	N/A
30	F	36	no	no	no	0	N/A
31	F′	37	no	no	no	0	N/A

**Table 2:** Type of genioplasty and adjuvant procedures performed in order to correct asymmetry and to feminize or masculinize lower third of the face.

Patient	Genioplasty	T-Genioplasty	Feminization	Masculinization	Symmetrization	Angle Resection	Angle Implants
1	no	yes	no	no	yes	no	no
2	yes	no	no	no	yes	no	no
3	no	yes	yes	no	yes	no	no
4	yes	no	no	yes	no	no	yes
5	yes	no	yes	no	yes	no	no
6	yes	no	no	no	yes	no	no
7	yes	no	no	yes	yes	no	yes
8	yes	no	no	no	yes	yes	no
9	yes	no	no	no	yes	no	no
10	yes	no	no	no	yes	no	no
11	yes	no	no	no	yes	no	no
12	no	yes	yes	no	yes	yes	no
13	no	yes	no	no	yes	yes	no
14	yes	no	no	no	yes	no	no
15	no	yes	no	no	yes	no	no
16	yes	no	no	no	yes	no	no
17	no	yes	yes	no	yes	no	no
18	yes	no	no	no	yes	no	no
19	no	yes	no	no	yes	no	no
20	no	yes	yes	no	no	yes	no
21	no	yes	yes	no	no	yes	no
22	no	yes	yes	no	no	yes	no
23	no	yes	yes	no	no	yes	no
24	no	yes	yes	no	no	yes	no
25	no	yes	yes	no	no	yes	no
26	no	yes	yes	no	no	yes	no
27	yes	no	no	yes	no	no	yes
28	yes	no	yes	no	no	no	no
29	no	yes	yes	no	no	no	no
30	no	yes	yes	no	no	yes	no
31	no	yes	yes	no	no	yes	no

**Table 3:** Types of osteotomies performed in the patients who underwent T-shape genioplasty.

Patient	T-Genioplasty	Type
1	yes	Horizontal (advancement)
3	yes	M-shape (advancement)
12	yes	Horizontal
13	yes	Horizontal
15	yes	M-shape (advancement)
17	yes	Horizontal (advancement)
19	yes	Horizontal
20	yes	Horizontal
21	yes	M-shape
22	yes	Horizontal
23	yes	Horizontal
24	yes	M-shape
25	yes	Horizontal
26	yes	Horizontal (setback)
29	yes	Horizontal
30	yes	Horizontal (advancement)
31	yes	Horizontal

**Table 4:** Adjuvant procedures performed 3–6 months after bone recontouring. DPF—deep-plane facelift, MACSL—Minimal Access Cranial Suspension Lift, CNL—central neck lift, Endoscopic—full endoscopic ponytail facelift.

Patient	Bichectomy	Liposuction	VASER	Surgical Neck Lift	Face Lift	Type
1	no	no	no	no	no	x
2	no	no	no	no	no	x
3	no	no	no	no	no	x
4	no	no	no	no	no	x
5	no	no	no	no	no	x
6	no	no	no	no	no	x
7	yes	yes	yes	no	no	x
8	yes	yes	yes	no	no	x
9	no	no	no	yes	yes	DPFL
10	no	yes	yes	no	no	x
11	no	no	no	no	no	x
12	yes	yes	yes	no	yes	MACSL
13	no	yes	yes	yes	no	CNL
14	no	yes	yes	yes	no	CNL
15	yes	yes	yes	yes	no	CNL
16	no	no	no	no	no	x
17	no	no	no	no	no	x
18	no	no	no	no	no	x
19	yes	no	no	no	no	x
20	yes	yes	yes	no	yes	Endoscopic
21	no	no	no	no	no	x
22	no	no	no	yes	yes	DPFL
23	yes	no	no	yes	yes	DPFL
24	no	no	no	yes	no	CNL
25	yes	yes	no	yes	yes	Endoscopic
26	no	no	no	no	no	x
27	no	no	no	no	no	x
28	no	no	no	no	no	no
29	no	yes	no	yes	no	CNL
30	no	yes	yes	no	no	x
31	yes	yes	yes	no	yes	DPFL

## Data Availability

Data are contained within the article.

## Supplementary Materials

The following supporting information can be downloaded at: https://www.mdpi.com/article/10.3390/medicina60010139/s1, Video S1.
